# Missing Data in Randomized Clinical Trials for Weight Loss: Scope of the Problem, State of the Field, and Performance of Statistical Methods

**DOI:** 10.1371/journal.pone.0006624

**Published:** 2009-08-13

**Authors:** Mai A. Elobeid, Miguel A. Padilla, Theresa McVie, Olivia Thomas, David W. Brock, Bret Musser, Kaifeng Lu, Christopher S. Coffey, Renee A. Desmond, Marie-Pierre St-Onge, Kishore M. Gadde, Steven B. Heymsfield, David B. Allison

**Affiliations:** 1 Department of Biostatistics, School of Public Health, University of Alabama at Birmingham, Birmingham, Alabama, United States of America; 2 BlueCross BlueShield of Tennessee, Chattanooga, Tennessee, United States of America; 3 Department of Epidemiology, School of Public Health, University of Alabama at Birmingham, Birmingham, Alabama, United States of America; 4 Division of Cardiovascular Disease, Department of Medicine, University of Alabama at Birmingham, Birmingham, Alabama, United States of America; 5 Merck & Co., Inc, Rahway, New Jersey, United States of America; 6 Division of Biostatistics and Bioinformatics, Comprehensive Cancer Center, University of Alabama at Birmingham, Birmingham, Alabama, United States of America; 7 New York Obesity Research Center, St. Luke's/Roosevelt Hospital & College of Physicians & Surgeons, New York, New York, United States of America; 8 Departments of Psychiatry, Duke University Medical Centre, Durham, North Carolina, United States of America; 9 The Research Triangle Research Institute, Research Triangle Park, North Carolina, United States of America; 10 Clinical Nutrition Research Center, University of Alabama at Birmingham, Birmingham, Alabama, United States of America; 11 Department of Nutrition Sciences, University of Alabama at Birmingham, Birmingham, Alabama, United States of America; University of Oxford, United Kingdom

## Abstract

**Background:**

Dropouts and missing data are nearly-ubiquitous in obesity randomized controlled trails, threatening validity and generalizability of conclusions. Herein, we meta-analytically evaluate the extent of missing data, the frequency with which various analytic methods are employed to accommodate dropouts, and the performance of multiple statistical methods.

**Methodology/Principal Findings:**

We searched PubMed and Cochrane databases (2000–2006) for articles published in English and manually searched bibliographic references. Articles of pharmaceutical randomized controlled trials with weight loss or weight gain prevention as major endpoints were included. Two authors independently reviewed each publication for inclusion. 121 articles met the inclusion criteria. Two authors independently extracted treatment, sample size, drop-out rates, study duration, and statistical method used to handle missing data from all articles and resolved disagreements by consensus. In the meta-analysis, drop-out rates were substantial with the survival (non-dropout) rates being approximated by an exponential decay curve (e^−λt^) where λ was estimated to be .0088 (95% bootstrap confidence interval: .0076 to .0100) and *t* represents time in weeks. The estimated drop-out rate at 1 year was 37%. Most studies used last observation carried forward as the primary analytic method to handle missing data. We also obtained 12 raw obesity randomized controlled trial datasets for empirical analyses. Analyses of raw randomized controlled trial data suggested that both mixed models and multiple imputation performed well, but that multiple imputation may be more robust when missing data are extensive.

**Conclusion/Significance:**

Our analysis offers an equation for predictions of dropout rates useful for future study planning. Our raw data analyses suggests that multiple imputation is better than other methods for handling missing data in obesity randomized controlled trials, followed closely by mixed models. We suggest these methods supplant last observation carried forward as the primary method of analysis.

## Introduction

“Well conducted clinical trials are the fastest and safest way to find improved treatments and preventions…” NIDDK [1].

Obesity is associated with and believed to cause adverse conditions such as cardiovascular disease, stroke, type 2 diabetes mellitus, certain forms of cancer [2], and decreased longevity [3]. It is estimated that over 50 million Americans are obese, and recent data show no decreases in prevalence [4]. Currently available treatments are only of moderate efficacy, and not all treatments work for all individuals. Thus, it is critical to identify and evaluate new alternative treatments for both efficacy and safety. Several important questions about how to best design, interpret, and analyze randomized controlled trials (RCTs) for obesity treatments remain unanswered (for video proceedings of an NIH-funded conference on this topic, see: http://main.uab.edu/Shrp/Default.aspx?pid=97738#schedule).

One of the most challenging aspects of obesity RCTs is the seemingly inevitable high rate of loss to follow-up (‘dropout’). A recent editorial from NIH scientists began by praising one of the largest, best-evaluated pharmaceutical obesity RCTs conducted but concluded its opening with the remark that “an overriding concern is the failure to obtain final weight measurements on about half of the randomized participants.” Such high losses to follow-up are not atypical and create several problems, including: (A) reduced statistical power; (B) potential loss of internal validity if data are not missing completely at random (MCAR); and (C) challenges in analyzing the resulting incomplete datasets.

It is difficult to evaluate the scope of this problem and the appropriateness of investigators' responses to it because there has been no formal quantitative integration of the published information on dropout rates (DORs) and which methods are most commonly used to accommodate missing data in obesity RCTs. Hence, meta-analysis was employed to extract and model DOR, while real raw data sets were used to evaluate the performance of statistical strategies for handling missing data. Although simulation studies and derivations of asymptotic properties of some available statistical methods for accommodating missing data in inferential testing are available, there is no guarantee that the conditions simulated or under which the asymptotic properties were derived effectively represent real data in terms of factors such as the presence of outliers, degrees of dropout, shape of marginal distributions (e.g., extent of non-normality), or covariance structure among observations. Therefore, the purpose of this project was to conduct two separate evaluations to estimate the scope of the problem. First, we conducted a meta-analysis of obesity RCTs. Second, we analyzed multiple real raw datasets through various missing data methodologies. The results of such analyses have implications for the design of future obesity RCTs, for the interpretation of the relative rigor of individual past and future obesity RCTs, and importantly, for the choice of statistical method for their analysis.

## Methods

### Quantitative synthesis of published research: DORs and methods used to accommodate them

#### Data Source

Published articles were retrieved using searches performed on: 1) electronic databases (MEDLINE and Cochrane database publications), 2) Cross-reference from original publications and review articles, and 3) manual searching of bibliographic references. We searched PubMed to identify publications for inclusion, imposing the following limits: date, RCTs, human studies, English language and peer-reviewed.

#### Inclusion Criteria

All studies used had to meet the following inclusion criteria: 1) the data were from human studies, 2) the study was an RCT, 3) the study reported DORs, 4) the study used one or more pharmaceuticals vs placebo, 5) weight loss and/or weight gain prevention was a study outcome, 6) the study was published in a peer-reviewed journal, 7) the study was published in the English language, and 8) the study was published between January 1, 2000 and December 31, 2006. One study (44) published in print in 2007 was included in our analysis because it showed up in our search in 2006 as an *epub*.

Multiple publication biases (including the same subjects reported in two or more papers) were avoided by carefully examining each study for duplication. All articles were double-checked independently for inclusion criteria by two of the authors (M.E. and O.T). Discrepancies were resolved by consensus. D. W. B. conducted final inclusion criteria verification (10%) on a random sample of the identified articles and obtained 100% agreement. One of three other authors (D.B.A, C.S.C., R.A.D) checked the coded information obtained from each article and again, discrepancies were resolved by consensus.

#### Study Searching

We divided our keyword search into four categories: 1) ‘obesity’ OR ‘weight loss’ OR ‘weight gain prevention’, which yielded 2111 studies, 2) sibutramine OR orlistat OR topiramate OR rimonabant OR recombinant leptin, which yield 286 studies, 3) combined categories 1 AND 2 of weight-related outcomes and pharmaceuticals, which yielded 199 studies, and 4) combined category 3 AND ‘placebo’, which yielded 141 studies. The 141 studies were further screened for inclusion and resulted in a final sample of 89 studies from our PubMed search. Secondly, we searched the Cochrane databases for meta-analyses of weight loss interventions using ‘weight loss’ and ‘obesity’ as keywords, which yielded 41 reviews of which 3 were reviews of pharmaceutical trials with weight loss or weight gain prevention as a major endpoint. Bibliographies of the Cochrane-derived studies were searched for publications eligible for inclusion. The search of all bibliographies yielded 32 additional studies for inclusion. Although this search was not expected to retrieve pharmaceutical obesity RCTs, it provided a sufficiently large sample to yield reasonably precise estimates of DORs as a function of study duration, which was our goal.

#### Data Extraction of study-level variables and results

Two reviewers (M.E. and O.T.) extracted the following data from all articles collected and resolved disagreements by consensus (21, 26–145; Appendix S1). The variables of interest included:

1) general information (authors and year of publication), 2) duration of the trial, 3) total sample size defined as the number of subjects randomized, 4) DORs defined as the total number of subjects that dropped out from the trial from the time of randomization to the time of completion, 5) methods used to accommodate missing data; e.g. completer's only, last observation carried forward (LOCF), mixed model (MM), and multiple imputation (MI), and 6) the specific drugs used for treatment.

#### Modeling DORs

In the meta-analyses for the i^th^ published article, the proportion of subjects remaining in the corresponding study and on whom a final endpoint measurement was obtained at time *t*, was recorded and denoted as 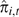. We then fit an exponential decay curve to these proportions using SPSS' non-linear regression and the model: 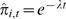, a simple model with a constant rate of drop-out over time. We solved for the value of λ by minimizing the sum of squared model residuals. Models were run both unweighted and weighted by the inverse of the variance for each observation. Standard errors, confidence intervals and p-values were obtained by bootstrapping with 1,000 bootstrap samples.

### Analysis of Multiple Real Raw Datasets to Evaluate Method Performance

#### Acquisition of RCT Raw Datasets

We obtained 12 real raw datasets from obesity RCTs conducted by (M.P.S., K.M.G., S.B.H., and D.B.A.) and one data set from the NIDDK data archive. All datasets were from an intervention for weight loss or weight gain prevention (Table 1).

**Table 1:** Real Datasets Acquired.

Study Number	Reference	Number Randomized	Number of completers	Duration (weeks)	Treatment	Number of post-baseline measurement points
1	RCT 1[17]	186	154	12	Herbal Supplement contain Ephedrine	6
2	RCT 2 [18]	102	87	12	Herbal Supplement contain Ephedrine	7
3	RCT 3 [19]	96	68	12	Acupressure device for weight loss	7
4	RCT 4 [20]	60	51	32	Zonisamide	6
5	RCT 5 [21]	30	21	12	Atomoxetine	5
6	RCT 6 [22]	75	47	12	Meal Replacement (Soy)	6
7	RCT 7 [23]	135	84	12	Herbal Supplement Contain Garcinia Cambogia	7
8	RCT 8 [24]	100	30	40	Meal Replacement (Soy)	3
9	RCT 9 [25]	100	58	12	Meal Replacement (Soy)	11
10	DPP [6]	2103	242	48 months	Metformin	9
11	NPY-1 [26]	206	159	12	Neuropeptide Y5R	5
12	NPY-1 [26]	1661	854	52	Neuropeptide Y5R	11

Abbreviations: RCT, randomized controlled trial; DPP, Diabetes Prevention Program; NPY-1, neuropeptide-Y-1.

#### Generation of Plasmode Datasets

A plasmode is a “numerical scientific example, preferably derived from data in a physical model, in which the relations generating measures are controlled and known to fit an explicit theoretical model” [5]. It is generally a ‘real’ data set in the sense that it is not a function of a computer simulation but has been obtained or manipulated in such a way that some aspect of the truth is known. Plasmodes constructed from real datasets have the advantage of real data in that they can be, by definition, realistic in terms of marginal distributions, covariance structures, presence of outliers, and patterns of dropout. Yet at the same time, they retain a key advantage of traditional simulations. Specifically, manipulation can be done so that some aspects of the data generating process are known with certainty. This allows one to empirically evaluate performance characteristics of analytic methods by determining the frequency with which a method obtains the *known* right answers.

We generated plasmodes under both the null and alternative hypotheses from the obtained 12 raw datasets. To generate plasmodes under the null hypothesis of no treatment effect on weight for each of the 12 datasets, we randomly permuted the treatment assignment indicators. This perfectly preserved the real data's marginal distributions, covariance structures, presence of outliers, and patterns of dropout, yet assured that all null hypotheses of no effect of treatment were true. However, it does not preserve any relation between missingness and treatment assignment. By analyzing such permuted datasets and observing the frequency that statistically significant results were obtained, we were able to evaluate whether our procedures were properly holding the type I error rate to the set level.

To generate plasmodes under the alternative hypothesis of some treatment effect on weight, constants were added to the body weights of each treatment group in each of the above randomly permuted plasmodes. The added constants were meant to mimic the treatment trajectory in Wadden et al. [6], which are trajectories common in obesity research. This essentially simulates data for power evaluation by imposing a treatment effect on the permuted datasets. The treatment effect was generated to have 50% power for the datasets in the LOCF condition. Power of 50% was chosen because at such middling levels it is relatively easy to see differences among methods in power that would not be easily apparent at very high power levels such as 90%. The LOCF condition was chosen for two reasons. First, it is in a sense a “complete dataset”, so when a dataset is analyzed with the missing values added back, one can see how much power has been lost. Second, generating a 50% power under the completer's only condition caused the power when analyzing the data under the LOCF condition to be as high as 100% in some dataset.

#### Statistical Analysis of Real & Plasmode RCT Datasets

Four different strategies for analyzing data with missing values were used to analyze the 12 real datasets and generated plasmodes. Plasmode simulations and all analyses of real and plasmode datasets were performed on SAS 9.1. With the exception of the intent-to-treat last observation carried forward (ITT-LOCF) method (defined below), patients in all of these methods had a baseline measurement and at least one post baseline measurement. Additionally, weight loss is calculated as the difference between weight at the end minus weight at the beginning of the trial. It should be noted that multiple imputation (MI), mixed model (MM), and completers only analysis (but not necessarily LOCF) will provide consistent parameter estimates (a consistent estimator is one that converges in probability to its estimate asymptotically in the sample size) if the missing values are MCAR. However, only MI and maximum likelihood (ML) will provide consistent parameter estimates when the missing values are missing at random (MAR), a less restrictive and more realistic situation (for further reading see Gadbury et. al. [7]).

#### Completers Only

In the completers only analysis, we used only the data for patients who came in for the baseline visit and the last follow up visit; that is, any patients who were missing any visits in the middle were still included.

#### Last Observation Carried Forward

In the LOCF analysis, if a subject's weight was missing at a visit, then the weight from the most proximal prior visit was used. For example, if a study has 5 visits and the participant only missed visit 3, then the value from visit 2 would be used as the participant's weight for visit 3. LOCF was conducted under two methods.

#### Intent- To- Treat Last Observation Carried Forward

This method preserved the most data in that it allowed for the possibility of carrying the baseline measurement forward to the end of the trial if a subject dropped out immediately after the baseline visit and before any follow up weights were taken. Therefore, it is possible to have some cases with only baseline measurements.

#### Last Observation Carried Forward

In this LOCF method, patients with only baseline measurements were not used. That is, all patients have a baseline and at least one post-baseline measurement of weight.

#### Multiple Imputation

MI is a missing data technique that imputes plausible values for the missing values. One generates *m* datasets with plausible values imputed for the missing values. Each of the *m* datasets is separately analyzed using the desired model (i.e. regression, ANOVA, etc.), generating *m* sets of parameter estimates. The *m* sets of parameter estimates are then combined using standard rules for MI analyses [8], [9]. The combined parameter estimates are then used for hypothesis testing and inference. For this study, the degrees of freedom for the combined parameter estimates were adjusted as outlined by Barnard and Rubin [10]. Additionally, only group membership (i.e., treatment or placebo) and measurements over time were used in the imputation process. Imputations were conducted using two methods.

#### MI for Monotone Missing Value

This imputation was conducted by first imputing enough data to impose a monotone missing data pattern on the original data via a Markov Chain Monte Carlo (MCMC) algorithm. A dataset with variables *X*_1_, *X*_2_, …, *X*_p_ has a monotone missing data pattern when *X_i_* is missing and subsequently *X_j_* for *j*>*I* is missing for a patient. If the missing data pattern was already monotone, then this step was skipped. Monotone missing data occurs frequently in longitudinal studies. The data were then imputed by assuming a monotone missing data pattern using the regression method proposed by Rubin [11].

#### MI for General Missing Data Pattern

In this imputation scheme, no assumption was made about the pattern of missing values except that they are MAR. The data were imputed via an MCMC algorithm with multiple chains and 1200 burn-in iterations. The MCMC algorithm used here is a two-step iterative process that begins by imputing plausible values for the missing values given the observed values in order to generate a complete data set [9]. Second, the complete data set is then used to compute the parameters of the posterior distribution. These parameters are then fed back into the first step to imputing plausible values for the missing values, which are then used in the next posterior step, etc. The process iterates long enough to reach the stationary or desired distribution, which in this case is multivariate normal.

#### Mixed Model

In this strategy, when a dataset has missing values all available data are used to directly estimate model parameters via ML. More specifically, restricted maximum likelihood (REML) was used in these applications. No participant is dropped from the analysis because all available data are used to obtain parameter estimates. The REML methods were conducted with a mixed model treating time as continuous or categorical and modeling ***V***, the variance of ***y***, in two ways (for further details see [12]). When time was treated as continuous, **V** was modeled as a function of the covariances of the random effects and random errors. In this particular case, the covariance of the random effects was unstructured, and the random errors were assumed independent and constant (homogenous). When time was treated as categorical, ***V*** was modeled as a function of the unstructured covariance of the random errors. The one exception was RCT 9. Large amount of missing data in RCT 9 led to unstable estimates with use of an unstructured covariance matrix, so unstructured covariances were replaced with autoregressive lag 1 [AR(1)] covariances when treating time as continuous or categorical.

## Results

### Scope of Missing Data Due to Dropouts

**Figure 1:**
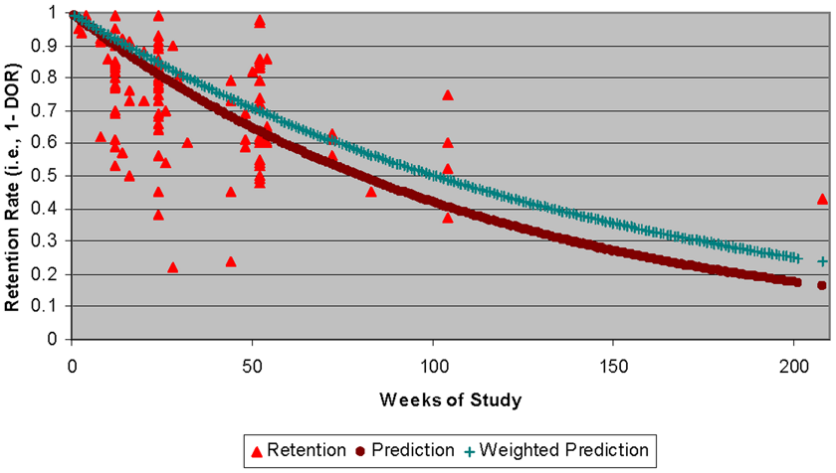
Scatter plot of dropouts over time with fitted exponential decay curve. Drop-out rates for six small (N = 18 to 60) studies that reported zero drop-outs were set to 1% to allow the analyses to proceed.

Our search identified 121 articles meeting inclusion criteria. The unweighted mean DORs of the 121 studies was 26.3%. DORs varied substantially among studies and, not surprisingly, as a function of study duration. The exponential function fitted to the meta-analysis data was statistically significant. In the unweighted analysis, the exponential coefficient (i.e., ‘hazard’) was .0088 (asymptotic p-value = 3.2*10^−28^; 95% bootstrap CI: .0076 to .0100) and in the weighted analysis was .0069. The data and the fitted curves are shown in Figure 1. As can be seen, at 1-year, we would expect 37% (SE≈1.76%) of patients to have dropped out. This curve can be used to crudely estimate dropout rates when planning future pharmaceutical studies. In doing so, it should be noted that the unweighted and weighted predictions are quite similar and appear to fit the data in an unbiased fashion through approximately 75 weeks. After that, these predictions diverge and appear a bit biased. Hence, their use in trials extending beyond 75 weeks is questionable, and more evidence from very long trials is needed.

#### Type of methods to adjust for attrition bias

**Figure 2:**
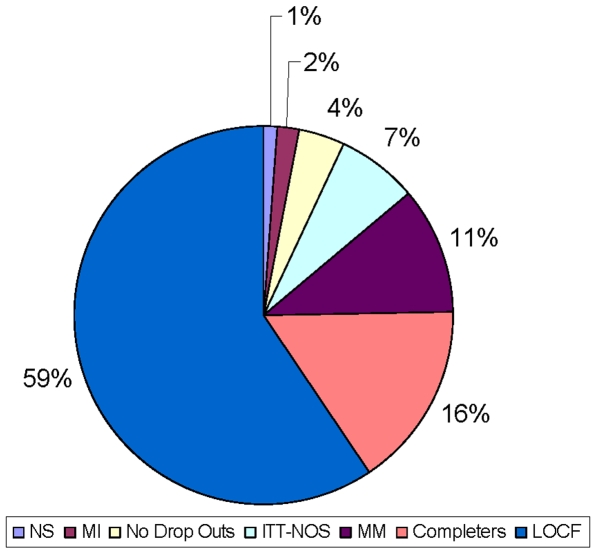
Percent of published studies using methods to accommodate drop-outs. For this chart, when an article reported using more than one analytic procedure, it was coded as having used the ‘best’ of the procedures it employed where the ranking was in ascending order: Completers only, LOCF (any variation on last observation carried forward); an unspecified intent to treat (ITT) analysis; any of several mixed model analyses (mixed), or multiple imputation (MI). ‘Completers’ denotes completer only analysis, ITT-NOS, ITT not otherwise specified, ‘No Drop Outs’, no dropouts reported, and NS, not specified.

Figure 2 displays the methods used to accommodate missing data in the published RCTs. As can be seen, it has now become the norm to do some type of intent-to-treat (ITT) analysis, though roughly 14% of studies still use only a completers analysis. The vast majority of studies that do ITT analyses use nothing more sophisticated that some variant of LOCF, and those few that do almost universally used some variation of a mixed model.

#### Performance of Methods

Table 2 displays the amount of observed and missing data points in each of the 12 real dataset. Table 3 displays mean differences (MD), and p-values for the actual analysis, type-1 error rates under the permutation-constructed nulls, and power for the ITT-LOCF, LOCF and completers only. Table 4 and 5 display the results for the MI methods. Lastly, Table 5 and 6 show the results of the Mixed Model methods treating time as continuous and categorical, respectively.

**Table 2:** Percent of Observed and Missing Data Points from the 12 Obesity RCT Datasets.

Intent-to-Treat				Baseline-Post-baseline			Imposed Treatment Mean
Data Set	Total Data Points	Observed Data Points	Proportion Missing	Total Data Points	Observed Data Points	Proportion Missing	Last Time Point
RCT 1	1116	1029	.08	1116	1029	.08	1.90
RCT 2	714	672	.06	714	672	.06	2.18
RCT 3	658	538	.18	644	536	.17	1.33
RCT 4	360	334	.07	348	332	.05	2.35
RCT 5	150	126	.16	130	122	.06	6.10
RCT 6	450	330	.27	354	314	.11	1.65
RCT 7	945	716	.24	833	700	.16	2.75
RCT 8	300	239	.20	249	222	.11	2.65
RCT 9	1089	586	.46	913	570	.38	2.30
RCT 10	18927	13133	.31	18639	13101	.30	0.64
RCT 11	1030	922	.10	1030	922	.10	0.23
RCT 12	18271	13344	.27	17105	13238	.23	0.66

Abbreviations: RCT, randomized controlled trial.

**Table 3:** Analysis of ITT-LOCF, LOCF, and completers for handling missing data in the 12 raw datasets using ordinary least squares.

	Actual Analysis			Null	Imposed Treatment Effect	
**ITT-LOCF**	Observed Mean Difference	Observed p-value	Permuted p-value	Empirical *α*	Mean Difference	Power
RCT 1^a^	4.07	1×10^−5^	<10^−5^	.0479	1.99	.539
RCT 2 a	2.60	.0103	.0113	.0522	2.13	.528
RCT 3	0.62	.2827	.2837	.0510	1.19	.515
RCT 4	5.01	<10^−5^	<10^−5^	.0505	2.26	.489
RCT 5	7.17	.0022	.0005	.0469	5.29	.482
RCT 6	0.03	.9551	.9561	.0510	1.17	.414
RCT 7	1.66	.2344	.2252	.0478	2.55	.429
RCT 8	1.66	.4133	.4270	.0485	3.71	.413
RCT 9	0.71	.3881	.3971	.0517	1.57	.443
RCT10	1.90	<10^−5^	<10^−5^	.0474	0.56	.537
RCT 11 a	1.30	.0009	.0006	.0531	0.82	.541
RCT 12	1.05	.0001	.0003	.0496	0.53	.481
**LOCF**						
RCT 1^a^	4.07	1×10^−5^	<10^−5^	.0479	1.99	.539
RCT 2 a	2.60	.0103	.0113	.0522	2.13	.528
RCT 3	0.61	.2964	.2976	.0509	1.21	.515
RCT 4	4.95	<10^−5^	<10^−5^	.0497	2.34	.502
RCT 5	9.12	.0005	<10^−5^	.0459	6.09	.511
RCT 6	0.39	.5906	.5868	.0498	1.50	.498
RCT 7	1.81	.2075	.2015	.0471	2.87	.508
RCT 8	2.09	.3731	.3825	.0471	4.48	.457
RCT 9	0.83	.3702	.3730	.0483	1.87	.500
RCT10	1.93	<10^−5^	<10^−5^	.0468	0.57	.537
RCT 11 a	1.30	.0009	.0006	.0531	0.82	.541
RCT 12	1.10	.0002	.0004	.0492	0.57	.484
**Completers**						
RCT 1	4.72	2×10^−5^	<10^−5^	.0474	1.90	.390
RCT 2	2.71	.0140	.0133	.0513	2.18	.480
RCT 3	0.65	.3767	.3759	.0523	1.34	.424
RCT 4	5.32	<10^−5^	<10^−5^	.0470	2.37	.451
RCT 5	9.34	.0038	.0011	.0484	6.12	.375
RCT 6	0.44	.6058	.6052	.0505	1.66	.468
RCT 7	2.14	.1880	.1842	.0475	2.74	.389
RCT 8	0.56	.8261	.8531	.0379	5.28	.512
RCT 9	1.58	.4435	.4403	.0512	2.27	.176
RCT10	0.88	.3411	.3497	.0500	0.65	.107
RCT 11	1.39	.0029	.0021	.0537	0.92	.490
RCT 12	1.42	.0030	.0038	.0500	0.66	.279

Abbreviations: ITT-LOCF, intent-to-treat-last observation carried forward; RCT, randomized controlled trial.

a Indicates missing data pattern is the same for ITT-LOCF and LOCF. Each permutation test is based on 10,000 permutations of each dataset.

**Table 4 pone-0006624-t004:** Analysis of ITT-LOCF, LOCF, and completers for handling missing data in the 12 raw datasets using Multiple Imputation.

	Actual Analysis			Null	Imposed Treatment Effect	
**MI (Monotone)**	Observed Mean Difference	Observed p-value	Permuted p-value	Empirical *α*	Mean Difference	Power
RCT 1	4.17	4×10^−5^	<10^−5^	0.0482	1.92	0.43
RCT 2	2.63	0.0161	0.0146	0.0483	2.18	0.468
RCT 3	0.92	0.2377	0.1688	0.0455	1.32	0.416
RCT 4	5.16	2×10^−5^	<10^−5^	0.0473	2.35	0.456
RCT 5	10.08	0.0006	<10^−5^	0.0453	6.02	0.42
RCT 6	0.47	0.582	0.74	0.0461	1.65	0.503
RCT 7	1.66	0.3014	0.2052	0.0469	2.75	0.399
RCT 8	2.34	0.3242	0.3821	0.0445	5.3	0.592
RCT 9	1.11	0.6768	0.8025	0.0354	2.28	0.205
RCT 10	1.43	0.0369	0.0481	0.0562	0.65	0.144
RCT 11	1.5	0.0011	0.0009	0.0503	0.93	0.486
RCT 12	1.13	0.0281	0.0168	0.0508	0.66	0.375
**MI (MCMC)**						
RCT 1	4.18	0.0006	0.0005	0.0506	1.92	0.431
RCT 2	3.06	0.0055	0.004	0.0488	2.18	0.473
RCT 3	0.91	0.1817	0.2277	0.0484	1.32	0.429
RCT 4	5.22	1×10^−5^	<10^−5^	0.0479	2.35	0.459
RCT 5	10.14	0.0005	<10^−5^	0.0478	6.02	0.428
RCT 6	0.3	0.7139	0.6776	0.0481	1.65	0.512
RCT 7	1.89	0.2236	0.1646	0.0492	2.75	0.402
RCT 8	2.22	0.3571	0.3628	0.044	5.3	0.589
RCT 9	1.96	0.2473	0.6461	0.0432	2.26	0.228
RCT 10	1.62	0.0399	0.0198	0.0568	0.65	0.145
RCT11	1.45	0.004	0.0029	0.0498	0.93	0.492
RCT 12	1.21	0.0035	0.0034	0.0506	0.66	0.376

Abbreviations: RCT, randomized controlled trial; MI, multiple imputation. Each permutation test is based on 10,000 permutations of each dataset.

**Table 5:** Mixed Models via Restricted Maximum Likelihood (RML) Treating Time as Continuous.

	Actual Analysis			Null	Imposed Treatment Effect	
**Mixed I**	Observed Estimate	Observed p-value	Permuted p-value	Empirical *α*	Estimate	Power
RCT 1	4.02	8×10^−5^	<10^−5^	0.0435	2.01	0.47
RCT 2	2.88	0.0056	0.0075	0.0602	1.97	0.455
RCT 3	0.79	0.2796	0.2626	0.0493	1.31	0.42
RCT 4	5	<10^−5^	<10^−5^	0.0454	2.41	0.488
RCT 5	8.99	0.0009	0.0004	0.0323	6.14	0.442
RCT 6	0.37	0.6285	0.6465	0.0569	1.75	0.605
RCT 7	2.44	0.121	0.1163	0.0468	2.37	0.315
RCT 8	2.3	0.3333	0.3339	0.0424	5.32	0.588
RCT 9	0.42	0.7923	0.8026	0.0561	2.2	0.285
RCT 10	1.61	0.0012	0.0064	0.0947	0.72	0.328
RCT 11	1.51	0.001	0.0006	0.0516	0.92	0.498
RCT 12	1.2	0.0038	0.002	0.0031	0.62	0.3
**Mixed II**						
RCT 1	4.78	<10^−5^	<10^−5^	0.0487	1.86	0.429
RCT 2	2.44	0.0176	0.0228	0.0604	1.61	0.34
RCT 3	0.85	0.2203	0.24	0.0662	1.34	0.475
RCT 4	4.73	1×10^−5^	<10^−5^	0.0602	2.61	0.617
RCT 5	9.28	0.0004	0.0003	0.0584	6.07	0.494
RCT 6	0.14	0.8394	0.8517	0.0676	2.21	0.84
RCT 7	2.37	0.1229	0.1354	0.0601	1.88	0.238
RCT 8	2.29	0.3327	0.3366	0.0462	5.32	0.6
RCT 9	0.45	0.6728	0.7853	0.1824	2.3	0.549
RCT 10	1.26	0.0161	0.0171	0.0567	0.75	0.303
RCT 11	1.47	0.0015	0.0013	0.0526	0.93	0.505
RCT 12	1.16	0.0025	0.0041	0.0542	0.62	0.365

Abbreviations: RCT, randomized controlled trial; I, modeling ***V*** (variance matrix of y) as a function of ***G*** (variance matrix of random effect) and ***R*** (random errors); II, modeling **V** = ***R***. Each permutation test is based on 10,000 permutations of each dataset.

**Table 6:** Mixed Models via Restricted Maximum Likelihood (RML) Treating Time as Categorical.

	Actual Analysis			Null	Imposed Treatment Effect	
**Mixed I**	Observed Estimate	Observed p-value	Permuted p-value	Empirical *α*	Estimate	Power
RCT 1	4.18	5×10^−5^	5×10^−5^	0.0424	1.89	0.422
RCT 2	2.87	0.0061	0.0061	0.063	2.17	0.514
RCT 3	0.73	0.2968	0.2883	0.0503	1.32	0.449
RCT 4	5.12	<10^−5^	<10^−5^	0.0467	2.35	0.458
RCT5	9.81	0.0005	0.0002	0.031	6.14	0.411
RCT 6	0.31	0.6811	0.7004	0.0617	1.65	0.557
RCT 7	2.03	0.2056	0.1926	0.0457	2.76	0.405
RCT 8	2.32	0.3297	0.3312	0.0428	5.32	0.588
RCT 9	0.18	0.9107	0.9129	0.0525	2.31	0.304
RCT 10	1.61	0.0071	0.0249	0.1031	0.63	0.241
RCT 11	1.45	0.0016	0.0012	0.0527	0.93	0.501
RCT 12	1.14	0.0068	0.0039	0.0345	0.66	0.333
**Mixed II**						
RCT 1	4.22	4×10^−5^	5×10^−5^	0.0455	1.89	0.433
RCT 2	2.8	0.0101	0.0061	0.052	2.17	0.486
RCT 3	0.82	0.2374	0.2883	0.0555	1.33	0.466
RCT 4	5.21	<10^−5^	<10^−5^	0.0506	2.35	0.474
RCT 5	9.86	0.0004	0.0002	0.048	6.14	0.464
RCT 6	0.3	0.6987	0.7004	0.0563	1.65	0.531
RCT 7	2.03	0.1925	0.1926	0.0504	2.76	0.428
RCT 8	2.32	0.3284	0.3312	0.0466	5.32	0.598
RCT 9	1.17	0.2681	0.9129	0.1786	2.32	0.557
RCT 10	1.28	0.042	0.0249	0.0565	0.64	0.183
RCT 11	1.42	0.0021	0.0012	0.0516	0.93	0.505
RCT 12	1.13	0.0034	0.0039	0.051	0.66	0.402

Abbreviations: RCT, randomized controlled trial; I, modeling ***V*** (variance matrix of y) as a function of ***G*** (variance matrix of random effect) and ***R*** (random errors); II, modeling **V** = ***R***. Each permutation test is based on 10,000 permutations of each dataset.

#### Performance with Actual Data

Two components were assessed with respect to the actual data. First, we examined whether the overall conclusions are affected by the choice of analysis method. Second, we examined how robust the conclusions were by comparing the p-values obtained for the standard t-test and the permutation test.

In general, regardless of the analysis method chosen, the overall conclusion of whether or not a significant effect was observed did not change if a result was deemed to be significant (i.e. the p-value was below the standard 5% level). The one exception was RCT 10 in which both completers only and Mixed II Cat (defined in Table 6) obtained non-significant results, whereas all other methods obtained significant results. This illustrates, among other things that the conventional wisdom that completers only analyses are liberal and that LOCF is conservative does not necessarily hold in all datasets.

As can be seen in Table 3, all of the permuted p-values were close to observed p-values, with the exception of RCT 8 under completers only in which the permutation test gave a slightly higher p-value. This suggests that the datasets considered in this work were very robust to the underlying assumptions of the t-test for these three approaches. The results in Table 4 suggest that the MI methods may not be quite as robust, with more noticeable differences observed between the permuted and observed p-values. The biggest differences were observed with RCT 9, which had a large amount of missing data. For the plasmode datasets derived from RCT 9, the observed p-values are noticeably smaller than the permuted p-values. However, the noticeably smaller observed p-values were still nowhere near significant and more importantly even for RCT 9, the empirical type-1 error rates when MI is used at the .05 alpha level were well preserved (see below). In contrast, for mixed models, Table 5 and 6 reveals that this same concern exists with RCT 9. However, in the case of mixed models with RCT 9 and RCT 10, the empirical type-1 error rates at the .05 alpha levels were not well preserved (see below). Hence, these results suggest that mixed model approaches should be viewed with skepticism in conditions similar to those prevailing in RCT 9 and RCT 10 which includes modest sample size (for RCT 9), a large proportion of missing data (for both RCT 9 and RCT 10), and a high ratio of measurement time-points to completing patients.

Because different approaches for analyzing data using mixed models were considered, we also compared the Akaike information criteria (AIC) and Bayesian information criteria (BIC) for the mixed models in order to see whether one of the methods led to a consistently better fit. AIC and BIC measure the goodness of fit of an estimated model and favor models that best explain the data using the fewest free parameters. Smaller AIC and BIC values indicate better fit. This analysis confirmed that treating time as a continuous variable is the preferred approach when there are many missing data coupled with many time points. Conversely, treating time as categorical better fits the data when there are fewer missing data and fewer time points.

#### Performance Under the Null

As can be seen in Tables 3–5, most of the empirical *α* values are close to .05, which is theoretically expected and desired. Four cases had values of empirical *α*<.05: RCT 8 under completers only, RCT 9 under MI (monotone), RCT 5 under Mixed I Cont., and RCT 5 under Mixed I Cat. Only the mixed model approaches led to some conditions with values of empirical α>.05, i.e., excess type-1 error rates. For certain conditions, both RCT 9 and RCT 10 had higher than expected empirical values under the null. These datasets represent two of the datasets with the largest amounts of missing data. Additionally, RCT 9 had one additional problem because an unstructured covariance could not be fit. Whenever this was attempted, the program gave either a covariance matrix that was not positive definite or an infinite likelihood. For that reason, we chose to impose an AR(1) covariance structure as has been done in prior studies. It is possible that the difficulty in specifying the covariance matrix with many time points and much missing data may contribute to the increased type I error rate.

These results suggest that, at least when missingness is unrelated to treatment assignment, all of the approaches we evaluated for handling missing data are adequate for protecting the desired type I error rate in the majority of realistic cases. However, mixed model test statistics are prone to increased type I error rates, particularly if utilized with large amounts of missing data. This is not too surprising since mixed model test statistics are based on asymptotic approximations, and others [13], [14] have raised concerns about inflated type I error rates when using these tests.

#### Performance Under the Alternative Hypothesis: Power

**Figure 3:**
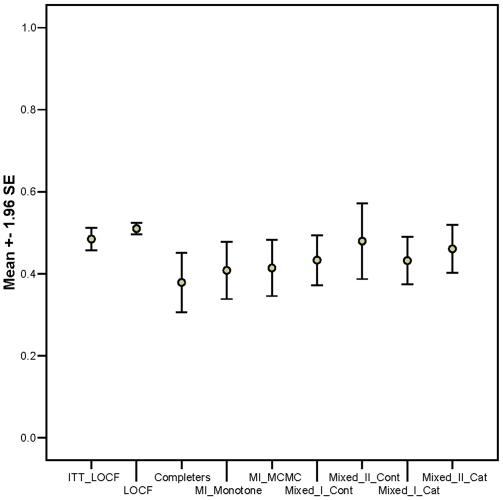
Mean power level averaged across all 12 RCT plasmode situations with 95% confidence intervals.

Tables 3–5 and Figure 3 summarize the results regarding statistical power. The completers only method has the least power and worsens with greater DOR. The LOCF methods have slightly greater power and less variability across datasets. This appears to be a function of simplicity and stability of the imputation process. Tables 4 and 5 suggest that the multiple imputation and mixed model approaches had comparable power, except in cases with substantial missing data (RCT 9 & RCT 10) where the mixed model approaches appear more powerful. Unfortunately, this apparent power advantage of mixed models in these two RCTs is not legitimate because the mixed models did not adequately hold the type 1 error rate in those situations.

## Discussion

Our quantitative survey of the literature on obesity RCTs shows that missing data are a very substantial problem. Moreover, the overwhelming majority of published reports use either completers only or LOCF techniques that have more stringent assumptions (i.e., completers only) or no theoretical foundation (i.e., LOCF) and are known to produce biased estimates in many circumstances. Reasons for this are likely manifold but may include skepticism on the part of many non-statistician (and some statistician) investigators' that the ‘fancier’ techniques such as mixed models and MI will produce reliable results with real data. Our results with the analyses of real data show that these more sophisticated and theoretically well-founded methods generally do not give wildly different results than the more primitive techniques. Moreover, in our plasmodes where the right answers are known yet the data distributions and amounts of missing data are realistic, MI and the mixed models performed well, except when there were very large amounts of missing data. These results should provide reassurance to applied investigators and journal editors and reviewers that these more sophisticated and theoretically-founded methods can be used in real obesity RCTs with reasonable confidence. That being said, when sample sizes are modest, many data points are missing, and the ratio of measurement points to patients is high, permutation tests should be encouraged when using MI or mixed model approaches to analyze weight loss data.

In interpreting our results, several limiting factors should be kept in mind. First, we only examined the performance of tests at alpha (type 1 error rate) levels of 0.05. This is a sensible choice because it seems to be the most commonly used alpha level in obesity RCTs. However, it is well known that statistical tests that depend on asymptotic properties, as do many of those that we evaluated, may perform well at higher alpha levels and be far less robust at lower alpha levels. Second, anecdotally, we are informed by several colleagues that since publication of the editorial by Simons-Morton [15], there has been a great increase in investigators' efforts to secure final weights on patients in obesity RCTs, even for patients who dropout of treatment. In contrast, this practice did not appear to be used in any of the trials we analyzed. Finally, we did not construct plasmode datasets in a manner that preserved any relationship between missing values and unobserved variables. This is because the nature of such relationships is not well-understood. We believe that studying such relations and incorporating models thereof into future plasmode or simulation studies of the kind we have conducted would be wise. Thus, generalization of our results to alpha levels less than .05, RCTs in which investigators secure final weights on patients in obesity RCTs even for patients who drop out of treatment, and studies with informative missingness must be made with caution.

### Implications for Study Design

To our knowledge, this is the first study to conduct a comprehensive analysis of DORs in obesity RCTs as a function of study duration. Landers effectively modeled subject retention in 12-week weight loss trial using survival analysis. The overall probability of completing that trial was 60% [16]. In our analysis of published pharmaceutical RCTs, the mean survival rate across 121 studies was 77.7%. Using study duration alone, we predicted that a study of 52 weeks would have a mean survival (retention) rate of 63%. The prediction equation (e^−.0088*weeks^) may prove helpful in determining needed sample size and estimating statistical power in future obesity RCTs that employ pharmaceutical agents. The extent to which this meta-analysis of DORs from pharmaceutical studies also applies to non-pharmaceutical weight loss studies remains open to question. Future research is also needed to examine the impact of study design and study-level patient characteristics on the prediction of DORs in obesity RCTS.

### Implications for Interpreting Past and Future Obesity RCTs

Our synthesis of DORs may also be helpful in interpreting individual RCTs. While we can always (justifiably) note anything less than perfect follow-up and complete data collection on all patients as a limitation in any RCT, knowing how that RCT fares relative to some norm helps put the magnitude of any accompanying criticism in perspective.

### Implications for Selection of Analytic Methods

It is well-established from theory that neither completers only analyses nor LOCF are guaranteed to return unbiased or consistent estimates of population effects even under conditions in which MI and mixed models will return consistent estimates. Thus, given that MI and mixed models are available, we could only see these *ad hoc* methods as justifiable as primary analytic strategies if empirical evidence showed MI and mixed models to perform poorly with data structures typical of obesity RCTs. We have now provided an evaluation of the possibility and found that MI and mixed models generally perform quite well with data structures typical of obesity RCTs. Therefore, we think that MI or mixed models should now be *de rigueur* in obesity RCTs with missing data.

This stands in contrast to the FDA's draft *Guidance for Industry Developing Products for Weight Management* which states “The analysis should be applied to the LOCF on treatment in the modified ITT population defined as patients who received at least one dose of study drug and have at least one post-baseline assessment of body weight. Sensitivity analyses employing other imputation strategies should assess the effect of dropouts on the results.” (See: http://www.fda.gov/cder/guidance/7544dft.pdf). We believe that our results coupled with established theory suggest that MI and mixed models should be methods of choice and LOCF and completers analysis used only as secondary or sensitivity analyses. That being said, our results do suggest caution in using mixed models when sample size is small, time points are many, and the proportion of data that are missing is high. In such situations, we recommend coupling mixed models with permutation testing for robustness. MI seems to have some robustness advantages over mixed models, and therefore we would recommend it as a method of choice. Additionally, MI does have other advantages. First, although not done for this study, MI can use as much of the data as possible by including other variables that are not explicitly in the model of interest. Second, once data have been imputed, the imputed data can be used to conduct a variety of analyses. Lastly, even if the MI model is incorrect, inferences made from the model of interest tend to remain valid [9]. That being said, the amount of data amassed herein suggesting the superiority of MI over mixed models in the context of RCTs is modest and further research is warranted.

## Supporting Information

Appendix S1 Pharmaceutical obesity RCTs used to evaluate the scope of the missing data problem.pdf
(0.27 MB DOC)
