# Supplementary material for: Missing Data in Randomized Clinical Trials for Weight Loss: Scope of the Problem, State of the Field, and Performance of Statistical Methods
Source: PLoS One. 2009 Aug 13;4(8):e6624. doi: 10.1371/journal.pone.0006624 (PMC2720539; doi:10.1371/journal.pone.0006624)
Supplement: Appendix S1 — Pharmaceutical obesity RCTs used to evaluate the scope of the missing data problem.pdf (0.27 MB DOC) [file pone.0006624.s001.doc]

**Appendix 1: Pharmaceutical obesity RCTs used to evaluate the scope of the missing data problem**

| **Reference (Year)** | **Duration of study (weeks)** | **No. of Subjects Randomized** | **Drop-outs** | **Drop-outs Percentage** | **Method used for handling drop-outs** | **Drug Used** |
| --- | --- | --- | --- | --- | --- | --- |
| Gadde et al., 2006 [21] | 12 | 30 | 9 | 30 | LOCF | Atomoxetine |
| Erondu et. al., 2006 [26] | 52 | 1661 | 832 | 50.09 | ITT-NOS |  |
| Berkowitz et. al., 2006 [27] | 52 | 498 | 137 | 27.51 | MM | Sibutramine |
| Garcia-Morales et. al., 2006 [28] | 24 | 51 | 11 | 21.57 | LOCF | Sibutramine |
| | Pi-Sunyer et. al., 2006 [29] | | --- | |  | | 104 | 3045 | 1924 | 63.19 | LOCF | Rimonabant |
| Lustig et. al., 2006[ 30] | 24 | 172 | 46 | 26.74 | LOCF | Octreotide |
| | Lord et. al., 2006 [31] | | --- | | 44 | 44 | 12 | 27.27 | ITT-NOS | Metformin |
| | Wirth et. al., 2006 [32] | | --- | | 12 | 195 | 32 | 16.41 | LOCF | Sibutramine |
| Bauer et al., 2006 [33] | 16 | 73 | 20 | 27.40 | Completers | Sibutramine |
| Kopelman et. al., 2006 [34] | 12 | 371 | 76 | 20.49 | LOCF | Cetilistat |
| Scheen et. al., 2006 [35] | 52 | 1047 | 155 | 14.8042 | LOCF, BOCF, MM | Rimonabant |
| Merideth 2006 [36] | 26 | 40 | 12 | 30 | LOCF | Lamotrigine |
| Maahs et. al., 2006 [37] | 24 | 40 | 6 | 15 | LOCF, Completers | Orlistat |
| Kim et. al., 2006 [38] | 12 | 68 | 32 | 47.06 | LOCF | Phentermine |
| O’Reardon et. al., 2006 [39] | 8 | 37 | 3 | 8.11 | MM | Sertraline |
| Zelber-Sagi et. al., 2006 [40] | 12 | 52 | 8 | 15.38 | LOCF | Orlistat |
| McElroy et. al., 2006 [41] | 16 | 60 | 30 | 50.00 | LOCF, MM | Zonisamide |
| Blonde et. al., 2006 [42] | 30 | 1446 | 321 | 22.20 | LOCF | Exenatide |
| Toplak et. al., 2006 [43] | 24 | 646 | 398 | 61.61 | LOCF | Topiramate |
| Kuo et. al., 2006 [44] | 12 | 60 | 0 | 0 | No drops | Orlistat |
| Silberstein et. al., 2006 [45] | 20 | 213 | 58 | 27.23 | LOCF | Topiramate |
| Loew et. al., 2006 [46] | 10 | 56 | 4 | 7.14 | LOCF | Topiramate |
| Mathus-Viliegen et. al., 2006 [47] | 52 | 42 | 12 | 28.57 | Completers | Sibutramine |
| Hung et. al., 2005 [48] | 24 | 48 | 0 | 0 | No Drops | Sibutramine |
| Wang et. al., 2005 [49] | 12 | 60 | 0 | 0 | No Drops | Sibutramine |
| Chanoine et. al., 2005 [50] | 54 | 539 | 190 | 35.25 | LOCF | Orlistat |
| Despres et. al., 2005 [51] | 52 | 1036 | 390 | 37.64 | LOCF | Rimonabant |
| Van Gaal et. al., 2005 [52] | 52 | 1507 | 587 | 38.95 | LOCF | Rimonabant |
| Gody-Matos et. al., 2005 [53] | 24 | 60 | 10 | 16.67 | LOCF, MM | Sibutramine |
| Mathus-Viliegen et. al., 2005 [54] | 72 | 189 | 70 | 37.04 | LOCF | Sibutramine |
| Zelissen et. al., 2005 [55] | 12 | 284 | 51 | 17.96 | LOCF | Recombinant Leptin |
| Shi et. al., 2005 [56] | 12 | 249 | 13 | 5.22 | LOCF, completers | Orlistat |
| Henderson et. al., 2005 [57] | 24 | 37 | 6 | 16.22 | ITT-NOS | Sibutramine |
| Swinburn et. al., 2005 [58] | 52 | 339 | 70 | 20.65 | LOCF, Completers | Orlistat |
| Faria et. al., 2005 [59] | 24 | 221 | 31 | 14.03 | Completers | Sibutramine |
| Tonstad et. al., 2005[ 60] | 28 | 531 | 413 | 77.78 | LOCF | Topiramate |
| Golay et. al., 2005 [61] | 24 | 89 | 18 | 20.22 | ITT-NOS | Orlistat |
| Franco et. al., 2005 [62] | 52 | 40 | 6 | 15 | ITT-NOS | Genotropin |
| Gambineri et. al., 2005 [63] | 28 | 20 | 2 | 10 | NS | Octreotide |
| Rodriguez-Moctezuma et. al., 2005 [64] | 8 | 23 | 2 | 8.695652 | Completers | Metformin |
| Douek et. al., 2005 [65] | 52 | 183 | 32 | 17.48634 | ITT-NOS | Metformin |
| Nickel et. al., 2005 [66] | 10 | 43 | 6 | 13.95 | Completers | Topiramate |
| Grilo et. al., 2005 [67] | 12 | 50 | 11 | 22 | LOCF, Completers | Orlistat |
| DeFronzo et. al., 2005 [68] | 30 | 336 | 64 | 19.05 | LOCF | Exenatide |
| Milano et. al., 2005 [69] | 12 | 20 | 0 | 0 | No Drops | Sibutramine |
| De Simone et. al., 2004 [70] | 12 | 29 | 9 | 31.03 | Completers | Sibutramine |
| Villareal et. al., 2004 [71] | 24 | 56 | 4 | 7.14 | LOCF | DHEA |
| Albert et al., 2004 [72] | 24 | 59 | 20 | 33.90 | LOCF, Completers | Recombinant |
| Torgerson et. al., 2004 [73] | 208 | 3305 | 1891 | 57.22 | LOCF, Completers | Orlistat |
| Astrup et. al., 2004 [74] | 44 | 561 | 308 | 54.90 | LOCF | Topiramate |
| Wilding et. al., 2004 [75] | 83 | 1289 | 709 | 55.00 | LOCF | Topiramate |
| Sanchez Reyes et. al., 2004 [76] | 52 | 86 | 39 | 45.35 | LOCF | Sibutramine |
| Guy-Grand et. al., 2004 [77] | 24 | 1004 | 89 | 8.76 | LOCF | Orlistat |
| Berne et. al., 2004 [78] | 52 | 221 | 31 | 14.03 | LOCF | Orlistat |
| Erdmann et. al., 2004 [79] | 24 | 384 | 82 | 21.35 | ITT-NOS | Orlistat |
| Kelly et. al, 2004 [80] | 24 | 52 | 13 | 25 | Completers | Orlistat |
| Kaukua et. al., 2004 [81] | 52 | 236 | 4 | 1.69 | LOCF | Sibutramine |
| Ersoz et. al., 2004 [82] | 12 | 57 | 22 | 38.60 | Completers | Sibutramine |
| Hainer et. al., 2004 [83] | 52 | 80 | 13 | 13 | Completers | Sibutramine |
| Tiikkainen et. al., 2004 [84] | 24 | 47 | 10 | 21.28 | Completers | Orlistat |
| Hoegar et. al., 2004 [85] | 48 | 38 | 15 | 39.47 | Completers | Metformin |
| Hauner et. al., 2004 [86] | 54 | 362 | 145 | 40.06 | LOCF | Sibutramine |
| Raskin et. al., 2004 [87] | 12 | 323 | 131 | 40.56 | LOCF | Topiramate |
| Hollander et. al., 2003 [88] | 52 | 656 | 308 | 46.95 | LOCF | Pramlitide |
| Barkeling et. al., 2003 [89] | 2 | 38 | 2 | 5.26 | Completers | Sibutramine |
| Appolinario et. al., 2003 [90] | 12 | 60 | 12 | 20 | MM | Sibutramine |
| Fanghanel et. al., 2003 [91] | 26 | 57 | 10 | 17.54 | LOCF, Completers | Sibutramine |
| Bray et. al., 2003 [92] | 24 | 385 | 128 | 33.25 | LOCF, Completers | Topiramate |
| **Ettinger et. al., 2003 [93] | 12 | 173 | 50 | 28.90 | LOCF, Completers | Recombinant |
| Gadde et. al., 2003 [94] | 32 | 60 | 24 | 40 | LOCF, Completers, MM | Zonisamide |
| Halpern et. al., 2003 [95] | 24 | 343 | 63 | 18.37 | LOCF | Orlistat |
| Krempf et. al., 2003 [96] | 78 | 696 | 271 | 38.94 | LOCF, Completers | Orlistat |
| Berkowitz et. al., 2003* [97] | 26 | 82 | 8 | 9.76 | Completers, MM | Sibutramine |
| Lustig et. al., 2003 [98] | 24 | 20 | 2 | 10 | MM | Octreotide |
| McNulty et. al., 2003 [99] | 52 | 195 | 50 | 25.77 | LOCF | Sibutramine |
| Tambascia et. al., 2003 [100] | 24 | 40 | 9 | 22.5 | Completers | Sibutramine |
| Zhi et. al., 2003 [101] | 3 | 32 | 2 | 6.25 | Completers | Orlistat |
| Lejeune et. al., 2003 [102] | 14 | 24 | 2 | 8.33 | Completers | Recombinant |
| Wulffele et. al., 2003 [103] | 16 | 390 | 37 | 9.487 | Completers | Metformin |
| McElory et. al., 2003 [104] | 14 | 61 | 26 | 42.62 | LOCF, MM, Completers | Topiramate |
| Derosa et. al., 2003 [105] | 52 | 99 | 3 | 3.03 | Completers | Orlistat, Fluvastatin, or Both |
| Cudkpwicz et. al., 2003 [106] | 52 | 296 | 134 | 45.27 | LOCF, MM, Imputation | Topiramate |
| Bergholm et. al., 2003 [107] | 26 | 57 | 10 | 17.54 | Completers | Orlistat |
| Hanefeld et. al., 2002 [108] | 48 | 383 | 119 | 31.07 | LOCF, Completers | Orlistat |
| Jain et. al., 2002 [109] | 26 | 422 | 193 | 45.73 | LOCF | Bupropion SR |
| Kelly et. al., 2002 [110] | 52 | 550 | 285 | 51.82 | LOCF | Orlistat |
| Miles et. al., 2002 [111] | 52 | 516 | 205 | 39.73 | LOCF | Orlistat |
| Anderson et. al., 2002 [112] | 48 | 327 | 135 | 41.28 | LOCF, MM, Completers | Bupropion SR |
| McMahon et. al., 2002 [113] | 52 | 220 | 100 | 45.45 | LOCF, Completers | Sibutramine |
| Sramek et. al., 2002 [114] | 12 | 61 | 6 | 9.84 | Completers | Sibutramine |
| Faria et. al., 2002 [115] | 24 | 109 | 23 | 21.10 | Completers | Sibutramine |
| Croft et. al., 2002 [116] | 44 | 423 | 320 | 75.65 | LOCF, Completers | Bupropion SR |
| Serrano- Rios et. al., 2002 [117] | 24 | 134 | 24 | 17.91 | LOCF | Sibutramine |
| Bakris et. al., 2002 [118] | 52 | 554 | 284 | 51.26 | LOCF | Orlistat |
| Ratner et. al, 2002 [119] | 52 | 538 | 157 | 29.18 | IIT | Pramlitide |
| Halpern et. al., 2002 [120] | 24 | 61 | 15 | 24.59 | LOCF, Completers | Sibutramine |
| Tong et. al., 2002 [121] | 24 | 60 | 4 | 6.67 | LOCF | Orlistat |
| Zannad et. al., 2002 [122] | 24 | 184 | 20 | 10.87 | LOCF | Sibutramine |
| Trouillot et. al., 2001 [123] | 4 | 23 | 0.00 | 0.00 | No drops | Orlistat |
| Wirth et. al., 2001 [124] | 44 | 1001 | 214 | 21.38 | LOCF, Completers | Sibutramine |
| ***Gadde et. al., 2001 [125] | 8 | 50 | 19 | 38 | LOCF | Bupropion SR |
| Smith et. al., 2001 [126] | 52 | 485 | 229 | 47.22 | LOCF | Sibutramine |
| Gokcel et. al., 2001 [127] | 24 | 60 | 6 | 10 | Completers | Sibutramine |
| Mules et. al., 2001 [128] | 24 | 294 | 35 | 11.90 | LOCF, Completers | Orlistat |
| Dujovne et. al., 2001 [129] | 24 | 322 | 102 | 31.68 | LOCF | Sibutramine |
| Hansen et. al., 2001 [130] | 72 | 467 | 204 | 43.68 | LOCF | Sibutramine |
| Storey et. al., 2001 [131] | 16 | 40 | 5 | 12.5 | Completers | Topiramate |
| Nam et. al., 2001 [132] | 12 | 18 | 0 | 0 | No Drops | Growth Hormone |
| Hazenberg, 2000 [133] | 12 | 127 | 21 | 16.54 | LOCF, Completers | Sibutramine |
| Hauptman et. al., 2000 [134] | 52 | 635 | 208 | 32.76 | LOCF, Completers | Orlistat |
| Fanghanel et. al., 2000 [135] | 24 | 109 | 25 | 22.94 | LOCF, Other | Sibutramine |
| Karhunen et. al., 2000 [136] | 52 | 96 | 6 | 6.25 | ITT-NOS, Completers | Orlistat |
| Lingarde et. al., 2000 [137] | 52 | 376 | 53 | 14.10 | ITT-NOS | Orlistat |
| Cuellar et. al., 2000 [138] | 24 | 69 | 38 | 55.07 | LOCF, Completers | Sibutramine |
| Finer et. al., 2000 [139] | 52 | 228 | 89 | 39.04 | LOCF, Completers | Orlistat |
| Fujioka et. al., 2000 [140] | 24 | 175 | 54 | 30.86 | LOCF, Completers | Sibutramine |
| Rössner et. al., 2000 [141] | 104 | 729 | 294 | 40.33 | LOCF, Completers | Orlistat |
| Wadden et. al., 2000 [142] | 16 | 34 | 8 | 23.529 | LOCF, Completers | Sibutramine, Orlistat |
| McMahon et. al., 2000 [143] | 52 | 224 | 104 | 46.43 | LOCF | Sibutramine |
| James et. al., 2000 [144] | 72 | 467 | 206 | 44.11 | LOCF, ITT | Sibutramine |
| Finer et. al., 2000 [145] | 12 | 91 | 8 | 8.79 | LOCF | Sibutramine |

*** Only the first 8 weeks were considered

** We excluded one of the randomized columns (39 subjects) since they were administered the drug and the placebo.

* Only the first 6 months were considered

BOCF: Baseline observation carried forward

LOCF: Any variation on LOCF that involved single imputation.

MM: Mixed model, not including mixed model of completers only data or mixed model analysis of data singly imputed with LOCF.

AUC: Area under the curve

ITT-NOS: Intent to treat analysis not otherwise specified

NS: Not specified

**References**

1. Berkowitz RI, Fujioka K, Daniels SR, et al (2006) Effects of sibutramine treatment in obese adolescents. *Ann Intern Med* 145: 81-90.
2. Garcia-Morales LM, Berber A, Macias-Lara CC, et al (2006) Use of sibutramine in Obese Mexican adolescents: A 6-month, randomized, double-blind, placebo-controlled, parallel-group trial. *Clin Ther* 28(5): 770-782.
3. Pi-Sunyer FX, Aronne LJ, Heshmati HM, Devin J, Rosenstock J; for the RIO-North America study group (2006) Effect of rimonabant, a Cannabinoid-1 receptor blocker, on weight and cardiometabolic risk factors in overweight or obese patients, RIO-North America: A randomized controlled trial. *JAMA* 295: 761-775.
4. Lustig RH, Greenway F, Velasquez-Mieyer P, et al (2006) A multicenter, randomized, double-blind, placebo-controlled, dose-finding trial of a long-acting formulation of octreotide in promoting weight loss in obese adults with insulin hypersecretion. *Int J Obes Relat Metab Disord* 30: 331-341.
5. Lord J, Thomas R, Fox B, Acharya U, Wilkin T (2006) The effect of metformin on fat distribution and metabolic syndrome in women with polycystic ovary syndrome-a randomized, double-blind, placebo-controlled trial. *BJOG* 113: 817-824.
6. Wirth A, Scholze J, Sharma AM, Matiba B, Boenner G (2006) Reduced left ventricular mass after treatment of obese patients with sibutramine: an echocardiographic multicentre study. *Diabetes Obes Metab* 8: 674-681.
7. Bauer G, Fischer A, Keller U (2006) Effect of sibutramine and of cognitive-behavioral weight loss therapy in obesity and subclinical binge eating disorder. *Diabetes Obes Metab* 8: 289-295.
8. Kopelman P, Bryson A, Hickling R, et al (2006) Cetilistat (ATL-962), a novel lipase inhibitor: a 12-week randomized, placebo-controlled study of weight reduction in obese patients. *Int J Obes Relat Metab Disord* 5: 1-6.
9. Scheen AJ, Finer N, Hollander P, Jansen MD, Van Gaal LF; for the RIO_Diabetes Study Group (2006) Efficacy and tolerability of rimonabant in overweight or obese patients with type 2 diabetes: a randomized controlled study. *Lancet* 368: 1660-72.
10. Meredith CH (2006) A single-center, double-blind, placebo-controlled evaluation of Lamotrigine in the treatment of obesity in adults. *J Clin Psychiatry* 67: 258-262.
11. Maahs D, de Serna DG, Kolotkin RL, et al (2006) Randomized, double-blind, placebo-controlled trial of orlistat for weight loss in adolescents. *Endocr Pract* 12(1): 18-28.
12. Kim KK, Cho HJ, Kang HC, et al (2006) Effects of weight reduction and safety of short-term phentermine administration in Korean obese people. *Yonsei M J* 47(5): 614-625.
13. O’Reardon JP, Allison KC, Martino NS, et al (2006) A randomized, placebo-controlled trial of sertraline in the treatment of night eating syndrome. *Am J Psychiatry* 163(5): 893-898.
14. Zelber-Sagi S, Kessler A, Brazowsky E, Webb M, et al (2006) A double-blind randomized placebo-controlled trial of orlistat for the treatment of nonalcoholic fatty liver disease. *Clin Gastroenterol Hepatol* 4: 639-644.
15. McElroy SL, Kotwal R, Guerdjikova AL, et al (2006) Zonisamide in the treatment of binge eating disorder with obesity: a randomized controlled trial. *J Clin Psychiatry* 67: 1897-1906.
16. Blonde L, Klein EJ, Han J, et al (2006) Interim analysis of the effects of exenatide treatment on A1C, weight and cardiovascular risk factors over 82 weeks in 314 overweight patients with type 2 diabetes. *Diabetes Obes Metab* 8: 436-447.
17. Toplak H, Hamann A, Moore R, et al (2007) Efficacy and safety of topiramate in combination with metformin in the treatment of obese subjects with type 2 diabetes: a randomized, double-blind, placebo-controlled study. *Int J Obes Relat Metab Disord* 31(1): 138-46.
18. Kuo CS, Pei D, Yao CY, et al (2006) Effect of orlistat in overweight poorly controlled Chinese female type 2 diabetic patients: a randomized, double-blind, placebo-controlled study. *Int J Clin Pract* 60(8): 906-910.
19. Silberstein SD, Hulihan J, Karim MR, et al (2006) Efficacy and tolerability of topiramate 200 mg/d in the prevention of migraine with/without aura in adults: a randomized, placebo-controlled, double-blind, 12-week pilot study. *Clin Ther* 28(7): 1002-1011.
20. Loew TH, Nickel MK, Muehlbacher M, et al (2006) Topiramate treatment for omen with borderline personality disorder: a double-blind, placebo-controlled study. *J Clin Psychopharmacol* 26(1): 61-66.
21. Mathus-Vlegen EM, van Ireland-van Leeuwen ML, Bennink RJ (2006) Influences of fat restriction and lipase inhibition on gastric emptying in obesity. *Int J Obes Relat Metab Disord* 30(8): 1203-1210.
22. Hung YJ, Chen YC, Pie D, et al (2005) Sibutramine improves insulin sensitivity without alteration of serum adiponectin in obese subjects with Type 2 diabetes. *Diabet Med* 22(8): 1024-1030.
23. Wang TF, Pei D, Li JC, et al (2005) Effects of sibutramine in overweight poorly controlled Chinese female type 2 diabetic patients: a randomized, double-blind, placebo-controlled study. *Int J Clin Pract* 59(7): 746-750.
24. Chanoine JP, Hampl S, Jensen G, et al (2005) Effect of orlistat on weight and body composition in obese adolescents. A randomized controlled trial. *JAMA* 293(23): 2873-2883.
25. Despres JP, Golay A, Sjostrom L; for the rimonabant in obesity-lipids study group (2005) Effects of rimonabant on metabolic risk factors in overweight patients with dyslipidemia. *N Engl J Med* 353(20): 2121-2134.
26. Van-Gaal LF, Rissanen AM, Scheen O, Zielger AJ, Rossner S; for the RIO-Study group (2005) Effects of cannabinoid-1 receptor blocker rimonabant on weight reduction and cardiovascular risk factors in overweight patients: 1-year from the RIO-Europe study. *Lancet* 365: 1389-97.
27. Godoy-Matos A, Carraro L, Vieira A, Oliverira J, et al (2005) Treatment of obese adolescents with sibutramine: A randomized, double-blind, controlled study. J *Clin Endocrinol Metab* 90(3): 1460-1465.
28. Mathus-Vilegen EMH; for the Balance study group (2005) Long-term maintenance of weight loss with sibutramine in a GP setting following a specialist guided very-low-calorie diet: a double-blind, placebo-controlled, parallel group study. *Eur J Clin Nutr* 59(S1): S31-S39.
29. Zelissen PMJ, Stenlof K, Lean MEJ, Fogteloo J, Keulen ETP, Wilding J, et al; on behalf of the author group (2005) Effect of three treatment schedules of recombinant methionyl human leptin on body weight in obese adults: a randomized, placebo-controlled trial. *Diabetes Obes Metab* 7: 755-761.
30. Shi Y-F, Pan CY, Hill J, Gao Y (2005) Orlistat in the treatment of overweight or obese Chinese patients with newly diagnosed type 2 diabetes. *Diabet Med* 22: 1737-1743.
31. Henderson DC, Copeland PM, Daley TB, et al (2005) A double-blind, placebo-controlled trial of sibutramine for Olanzapine- associated weight gain. *Am J Psychiatry* 162: 954-962.
32. Swinburn BA, Carey D, Hills AP, et al (2005) Effect of orlistat on cardiovascular disease risk in obese adults. *Diabetes Obes Metab* 7: 254-262.
33. Faria AN, Filho FFR, Kohlmann NE, Ferreira SRG, Zanella MT (2005). Effects of sibutramine on abdominal fat mass, insulin resistance and blood pressure in obese hypersensitive patients. *Diabetes Obes Metab* 7: 246-256.
34. Tonstad S, Tykarski A, Weissgarten J, Ivleva A, Levy B, Kumar A, et al; for the OBHT-001 study group (2005) Efficacy and safety of topiramate in the treatment of obese subjects with essential hypertension. *Am J Cardiol* 96: 243-251.
35. Golay A, Laurent-Jaccard A, Habicht F, et al (2005) Effect of orlistat in obese patients with binge eating disorder. *Obes Res* 13(10): 1701-1708.
36. Franco C, Brandberg J, Lonn L, et al (2005) Growth hormone treatment reduces abdominal visceral fat in postmenopausal women with abdominal obesity: A 12-month placebo-controlled trial. *J Clin Endocrinol Metab* 90(3): 1466-1474.
37. Gambineri A, Patton L, Iasio RD, et al (2005) Efficacy of octreotide-LAR in dieting women with abdominal obesity and polycystic ovary syndrome. *J Clin Endocrinol Metab* 90: 3854-3862.
38. Rodinguez-Moctezuma JR, Lopez GR, Carmona JML, Rosas MJG (2005) Effects of metformin on the body composition in subjects with risk factors for type 2 diabetes. *Diabetes Obes Metab* 7: 189-196.
39. Douek IF, Allen SE, Ewingst P, Gale EAM, Bingley PJ; for the Metformin Trial Group (2005) Continuing metformin when starting insulin in patients with type 2 diabetes: a double-blind randomized placebo-controlled trial. *Diabet Med* 22: 634-640.
40. Nickel C, Lahmann C, Tritt K, et al (2005) Topiramate in treatment of depressive and anger symptoms in female depressive patients: a randomized, double-blind, placebo-controlled study. *J Affect Disord* 87: 243-252.
41. Grilo CM, Masheb RM, Salant SL (2005) Cognitive behavioral therapy guided self-help and orlistat for the treatment of binge eating disorder: a randomized, double-blind, placebo-controlled trial. *Biol Psychiatry* 57: 1193-1201.
42. DeFronzo RA, Ratener RE, Han J, et al (2005) Effects of Exenatide (Exendin-4) on glycemic control and weight over 30 weeks in metformin-treated patients with type 2 diabetes. *Diabetes Care* 28(5): 1092-1100.
43. Milano W, Petrella C, Casella A, et al (2005) Use of sibutramine, an inhibitor of the reuptake of serotonin and noradrenaline, in the treatment of binge eating disorder: a placebo-controlled study. *Adv Ther* 22(1): 25-31.
44. De Simone G, Romano C, De Caprio C, et al (2005) Effects of sibutramine-induced weight loss on cardiovascular system in obese subjects. *Nutr Metab Cardiovasc Dis* 15(1): 24-30.
45. Villareal DT, Holloszy JO (2004) Effect of DHEA on abdominal fat and insulin action in elderly women and men. *JAMA* 292(18): 2243-2248.
46. Albert SG, Mooradian AD (2004) Low-dose recombinant human growth hormone as adjuvant therapy to lifestyle modifications in the management of obesity. *J Clin Endocrinol Metab* 89(2): 695-701.
47. Torgerson JS, Hauptman J, Boldrin MA, Sjostrom L (2004) XENical in the prevention of diabetes in obese subjects (XENDOS) study. A randomized study of orlistat as an adjunct to lifestyle changes for the prevention of type 2 diabetes in obese patients. *Diabetes Care* 27(1): 155-161.
48. Astrup A, Caterson J, Zelissen P, Guy-Grand B, Carruba M, Levy B, Sun X, et al; for the OBES-004 study group (2004) Topiramate: Long-tern maintenance of weight loss induced by a low-calorie diet in obese subjects. *Obes Res* 12(10): 1658-1669.
49. Wilding J, Van Gaal L, Rissanen A, Vercruysse F, Fitchet M; for the OBES-002 study group (2004) A randomized double-blind placebo-controlled study of the long-term efficacy and safety of topiramate in the treatment of obese subjects. *Int J Obes Relat Metab Disord* 28: 1399-1410.
50. Sanchez-Reyes L, Fanghanel G, Yamamoto J, et al (2004) Use of sibutramine in overweight adult Hispanic patients with type 2 diabetes mellitus: a 12-month, randomized, double-blind, placebo-controlled clinical trial. *Clin Ther* 26(9): 1427-1435.
51. Guy-Grand B, Drouin P, Eschwege E, et al (2004) Effects of orlistat on obesity-related diseases-a six-month randomized trial. *Diabetes Obes Metab* 6: 375-383.
52. Berne C; on behalf of the orlistat Swedish type 2 diabetes study group (2004) A randomized study of orlistat in combination with a weight management programme in obese patients with type 2 diabetes treated with metformin. *Diabet Med* 22: 612-618.
53. Erdmann J, Lippl F, Klose G, Schusdziarra V (2004) Cholesterol lowering effect of dietary weight loss and orlistat treatment-efficacy and limitations. *Aliment Pharmacol Ther* 19: 1173-1179.
54. Kelly DE, Kuller LH, McKolanis TM, et al (2004) Effects of moderate weight loss and orlistat on insulin resistance, regional adiposity, and fatty acids in type 2 diabetes. *Diabetes Care* 27(1): 33-40.
55. Kaukua JK, Pekkarinen TA, Rissanen AM (2004) Health-related quality of life in a randomized placebo-controlled trial of sibutramine in obese patients with type II diabetes. *Int J Obes Relat Metab Disord* 28: 600-605.
56. Eroz HO, Ukinc K, Baykan M, et al (2004) Effect of low-dose metoprolol in combination with sibutramine therapy in normotensive obese patients: a randomized controlled study. *Int J Obes Relat Metab Disord* 28: 378-383.
57. Hainer V, Kunesova M, Bellisle F, Hill M, Braunerova R, Wagenknecht M; the STO study group (2004) Psychobehavioral and nutritional predictors of weight loss in obese women treated with sibutramine. *Int J Obes Relat Metab Disord* 29: 208-216.
58. Tiikkainen M, Bergholm R, Rissamen A, et al (2004) Effects of equal weight loss with orlistat and placebo on body fat and serum fatty acid composition and insulin resistance in obese women. *Am J Clin Nutr* 79: 22-30.
59. Hoegar KM, Kochman L, Wixom N, et al (2004) A randomized, 48-week, placebo-controlled trial of intensive lifestyle modification and/or metformin therapy in overweight women with polycystic ovary syndrome: a pilot study. *Fertil Steril* 82: 421-429.
60. Hauner H, Meier M, Wendland G, Kurscheid T, Lauterbach K, Study Group SA; SAT Study (2004) Weight reduction by sibutramine in obese subjects in primary care medicine: the SAT study. *Exp Clin Endocrinol Diabetes* 112(4): 201-207.
61. Raskin P, Donofrio PD, Rosenthal NR, et al (2004) Topiramate vs placebo in painful diabetic neuropathy: analgesic and metabolic effects. *Neurology* 63: 865-873.
62. Hollander PA, Levy P, Fineman MS, et al (2003) Pramlintide as an adjunct to insulin therapy improves long-term glycemic and weight control in patients with type 2 diabetes. *Diabetes Care* 26(3): 784-790.
63. Barkeling B, Elfhag K, Rooth P, Rössner S (2003) Short-term of sibutramine (Reductil™) on appetite and eating behavior and the long-term therapeutic outcome. *Int J Obes Relat Metab Disord* 27: 693-700.
64. Appolinario JC, Bacaltchuk J, Sichieri R, et al (2003) A randomized, double-blind, placebo-controlled study of sibutramine in the treatment of binge-eating disorder. *Arch Gen Psychiatry* 60: 1109-1116.
65. Fanghanel G, Cortinas L, Sanchez-Reyes L, et al (2003) Safety and efficacy of sibutramine in overweight Hispanic patients with hypertension. *Adv Ther* 20(2): 101-113.
66. Bray GA, Hollander P, Klein S, Kushner R, Levy B, Fitchet M; for the US Topiramate Research group (2003) A 6-month randomized, placebo-controlled, dose-ranging trial of topiramate for weight loss in obesity. *Obes Res* 11(6): 722-733.
67. Ettinger MP, Littlejohn TW, Schwartz SL, et al (2003) Recombinant variant of ciliary neurotrophic factor for weight loss in obese adults. A randomized, dose-ranging study. *JAMA* 289: 1826-1832.
68. Gadde KM, Franciscy DM, Wanger HR, Krishnan KRR (2003) Zonisamide for weight loss in obese adults: A randomized controlled trial. *JAMA* 289(14): 1820-1825.
69. Halpern A, Mancini MC, Suplicy H, et al (2003) Latin-American trial of orlistat for weight loss and improvement in glycemic profile in obese diabetic patients. *Diabetes Obes Metab* 5: 180-188.
70. Krempf M, Louvet JP, Allanic H, et al (2003) Weight reduction and long-term maintenance after 18 months treatment with orlistat of obesity. *Int J Obes Relat Metab Disord* 27: 591-597.
71. Berkowitz RI, Wadden TA, Tershakovec AM, Cronquist JL (2003) Behavior therapy and sibutramine for the treatment of adolescent obesity, a randomized controlled trial. *JAMA* 289(14): 1805-1812.
72. Lustig RH, Hinds PS, Ringwald-Smith K, et al (2003) Octreotide therapy of Pediatric hypothalamic obesity: A double-blind, placebo-controlled trial. *J Clin Endocrinol Metab* 88(6): 2586-2592.
73. McNulty SJ, Ehud UR, Gareth Williams; for the multicenter sibutramine study group (2003) A randomized trial of sibutramine in the management of obese type 2 diabetic patients treated with metformin. *Diabetes Care* 26(1): 125-131.
74. Tambascia MA, Geloneze B, Repetto EM, et al (2003) Sibutramine enhances insulin sensitivity ameliorating metabolic parameters in a double-blind, randomized, placebo-controlled trial. *Diabetes Obes Metab* 5: 338-344.
75. Zhi J, Moore R, Kanitra L (2003) The effect of short-term (21-days) orlistat treatment on the physiologic balance of six selected macrominerals and microminerals in obese adolescents. *J Am Coll Nutr* 22(5): 357-362.
76. Lejeune MPGM, Hukshorn CJ, Saris WHM, Westerterp-Plantenga MS (2003) Effect of dietary restraint during and following pegylated recombinant leptin (PEG-OB) treatment of overweight men. *Int J Obes Relat Metab Disord* 27: 1494-1499.
77. Wulffele MG, Kooy A, Lehert P, et al (2003) Effects of short-term treatment with metformin on serum concentrations of homocysteine, folate and vitamin B12 in type 2 diabetes mellitus: a randomized, placebo-controlled trial. *J Intern Med* 254: 455-463.
78. McElroy SL, Arnold LM, Shapira NA, et al (2003) Topiramate in the treatment of binge eating disorder associated with obesity: A randomized, placebo-controlled trial. *Am J Psychiatry* 160: 255-261.
79. Derosa G, Mugellini A, Ciccarelli L, Fogari R (2003) Randomized, double-blind, placebo-controlled comparison of the action of orlistat, fluvastatin, or both on anthropometric measurements, blood pressure, and lipid profile in obese patients with Hypercholesterolemia prescribed a standardized diet. *Clin Ther* 25: 1107-1122.
80. Cudkowica ME, Shefner JM, Schoenfeld DA, et al, Northeast ALS Consortium (2003) A randomized, placebo-controlled trial of topiramate in amyotrophic lateral sclerosis. *Neurology* 61(4): 456-464.
81. Bergholm R, Tiikkainen M, Vehkavaara S, et al (2003) Lowering LDL cholesterol rather than moderate weight loss improves endothelium-dependent vasodilatation in obese women with previous gestational diabetes. *Diabetes Care* 26(6): 1667-16672.
82. Hanefeld M, Sachse G (2002) The effects of orlistat on body weight and glycaemic control in overweight patients with type 2 diabetes: a randomized, placebo-controlled trial. *Diabetes Obes Metab* 4: 415-423.
83. Jain AK, Kaplan RA, Gadde KM, et al (2002) Bupropion SR vs. placebo for weight loss in obese patients with depressive symptoms. *Obes Res* 10(10): 1049-1056.
84. Kelly DE, J Hill, GA Bray, J Miles, et al (2002) Clinical efficacy of orlistat therapy in overweight and obese patients with insulin-treated type 2 diabetes. *Diabetes Care* 25(6): 1033-1041.
85. Miles JM, Doyle M, Letter L, et al (2002) Effect of orlistat in overweight and obese patients with type 2 diabetes treated with metformin. *Diabetes Care* 5(7): 1123-1128.
86. Anderson JW, Greenway FL, Fujioka K, et al (2002) Bupropion SR enhances weight loss: A 48-week double-blind, placebo-controlled trial. *Obes Res* 10(7): 633-641.
87. McMahon FG, Weinstein SP, Rowe E, Ernest KR, Johnson F, Fujioka K; the sibutramine in hypertensive clinical study group (2002) Sibutramine is safe and effective for weight loss in obese patients whose hypertension is well controlled with angiotension-converting enzyme inhibitors. *J Hum Hypertens* 16: 5-11.
88. Sramek JJ, Leibowitz MT, Weinstein SP, et al (2002) Efficacy and safety of sibutramine for weight loss in obese patients with hypertension well controlled by β-adrenergic blocking agent: a placebo-controlled, double-blind, randomized trial. *J Hum Hypertens* 16: 13-19.
89. Faria AN, Filho FFR, Lerario DDG, et al (2002) Effects of sibutramine on the treatment of obesity in patients with arterial hypertension. *Arq Bras Cardiol* 78(2): 176-180.
90. Croft H, Houser TL, Jamerson BD, et al (2002) Effect on body weight of bupropion sustained-release in patients with major depression treated for 52 weeks. *Clin Ther* 24(4): 662-672.
91. Serrano-Rios M, Melchionda N, Moreno-Carreterot E (2002) Role of sibutramine in the treatment of obese type 2 diabetic patients receiving sulphonylurea therapy. *Diabet Med* 19: 119-124.
92. Bakris G, Calhoun D, Egan B, Hellmann C, Dolker M, Kingma I; on behalf of the orlistat and resistant hypertension investigators (2002) Orlistat improves blood pressure control in obese subjects with treated but inadequately controlled hypertension. *Journal Hypertens* 20: 2257-2267.
93. Ratner RE, Want LL, Fineman MS, et al (2002) Adjunctive therapy with the amylin analogue pramlitide leads to a combined improvement in glycemic and weight control in insulin-treated subjects with type 2 diabetes. *Diabetes Technol Ther* 4(1): 51-61.
94. Halpern A, Leite CC, Herszkowicz N, Barbato A, Costa APA (2002) Evaluation of efficacy, reliability, and tolerability, of sibutramine in obese patients, with an echocardiographic study. *Rev Hosp Clin Fac Med S Paulo* 57(3): 98-102.
95. Tong PCY, Lee ISK, Sea MM, et al (2002) The effect of orlistat-induced weight loss, without concomitant hypocaloric diet, on cardiovascular risk factors and insulin sensitivity in young obese Chinese subjects with or without type 2 diabetes. *Arch Intern Med* 162: 2428-2435.
96. Zannad F, Gille B, Grentzinger A, et al (2002) Effects of sibutramine on ventricular dimensions and heart valves in obese patients during weight reduction. *Am Heart J* 144: 508-515.
97. Trouillot TE, Pace DG, McKinley C, et al (2001) Orlistat maintains biliary lipid composition and hepatobiliary function in obese subjects undergoing moderate weight loss. *Am J Gastroenterol* 96(6): 1888-1894.
98. Wirth A, Krause J (2001) Long-term weight loss with sibutramine: a randomized controlled trial. *JAMA* 286(11): 1331-1339.
99. Gadde KM, Parker CB, Maner LG, et al (2001) Bupropion for weight loss: An investigation of efficacy and tolerability in overweight and obese women. *Obes Res* 9(9): 544551.
100. Smith IG on behalf of the members of the sibutramine clinical study 1047 team, Michael AG (2001) Randomized placebo-controlled trial of long-term treatment with sibutramine in mild to moderate obesity. *J Fam Pract* 50(6): 505-512.
101. Gokcel A, Tanaci N, Karakose H, et al (2001) Effects of sibutramine in obese female subjects with type 2 diabetes and poor blood glucose control. *Diabetes Care* 24: 1957-1960.
102. Muls E, Kolanowski J, Scheen A, Van-Gaal L; for the ObelHyx study group (2001) The effects of orlistat on weight and on serum lipids in obese patients with hypercholesterolemia: a randomized, double-blind, placebo-controlled, multicentre study. *Int J Obes Relat Metab Disord* 25: 1713-1721.
103. Dujovne CA, Zavoral JH, Rowe E, Mendal CM; for the sibutramine study group (2001) Effects of sibutramine on body weight and serum lipids: A double-blind, randomized, placebo-controlled study in 322 overweight and obese patients with dyslipidemia. *Am Heart J* 142: 489-497.
104. Hansen DL, Astrup A, Toubro S, Finer N, Kopelman P, Hilsted J, et al; for the study group (2001) Predictors of weight loss and maintenance during 2 years of treatment by sibutramine in obesity. Results from the European multi-centre STORM trial. *Int J Obes Relat Metab Disord* 25: 496-501.
105. Storey JR, Calder CS, Hart DE, Potter DL (2001) Topiramate in migraine prevention: a double-blind, placebo-controlled study. *Headache* 41(10): 968-975.
106. Nam SY, Kim KR, Cha BS, Song YD, Lim SK, Huh KB (2001) Low-dose growth hormone treatment combined with diet restriction decreases insulin resistance by reducing visceral fat and increasing muscle mass in obese type 2 diabetic patients. Int J Obes Relat Metab Disord 25: 1101-1107.
107. Hazenburg BP (2000) Randomized, double-blind, placebo-controlled, multicenter study sibutramine in obese hypertensive patients. *Cardiology* 94: 152-158.
108. Hauptman J, Lucas C, Boldrin MN, Collins H; for the orlistat group (2000) Orlistat in the long-term treatment of obesity in primary care settings. *Arch Fam Med* 9: 160-167.
109. Fanghanel G, Cortinas L, Sanchez-Reyes L, Berber A (2000) A clinical trail of the use of sibutramine for patients suffering essential obesity. *Int J Obes Relat Metab Disord* 24: 144-150.
110. Karhunen L, Franssila-Kallunki A, Rissanen P, et al (2000) Effect of orlistat treatment on body composition and resting energy expenditure during a two-year weight-reduction programme in obese Finns. *Int J Obes Relat Metab Disord* 24: 1567-1572.
111. Lindgrade F; on behalf of the orlistat Swedish multimorbidity study group (2000) The effect of orlistat on body weight and coronary heart disease risk profile in obese patients: The Swedish multimorbidity study. *J Intern Med* 248: 245-254.
112. Cueller GEM, Ruiz AM, Monsalve MCR, Berber A (2000) Six-month treatment of obesity with sibutramine 15 mg; A double-blind, placebo-controlled monocenter clinical trial in a Hispanic population. *Obes Res* 8(1): 71-82.
113. Finer N, James WPT, Kopelman PG, Lean MEJ, Williams G (2000) One-year treatment of obesity: a randomized, double-blind, placebo-controlled, multicentre, study of orlistat, a gastrointestinal lipase inhibitor. *Int J Obes Relat Metab Disord* 24: 306-313.
114. Fujioka K, Seaton TB, Rowe E, Jelinek CE, Raskin P, Lebovitz HE, et al; the Sibutramine/diabetes clinical study group (2000) Weight loss with sibutramine improves glycaemic control and other metabolic parameters in obese patients with type 2 diabetes mellitus. *Diabetes Obes Metab* 2: 175-187.
115. Rössner S, Sjöström L, Noack R, Meinders AD, Noseda G; on behalf of the European orlistat obesity study group (2000) Weight loss, weight maintenance, and improved cardiovascular risk factors after 2 years treatment with orlistat for obesity. *Obes Res* 8(1): 49-61.
116. Wadden TA, Berkowitz RI, Womble LG, et al (2000) Effects of sibutramine plus orlistat in obese women following 1 year of treatment by sibutramine: a placebo-controlled trial. *Obes Res* 8(6): 431437.
117. McMahon FG, Fujioka K, Singh BN, et al (2000) Efficacy and safety of sibutramine in obese white and African American patients with hypertension. *Arch Intern Med* 160: 2185-2191.
118. James WPT, Astrup A, Finer N, Hilsted J, Kopelman P, Rössner S, et al; for the STORM study group (2000) Effect of sibutramine on weight maintenance after weight loss: a randomized trial. *Lancet* 357: 2119-2125.
119. Finer N, Bloom SR, Frost GS, Banks LM, Griffiths J (2000) Sibutramine is effective for weight loss and diabetic control in obesity with type 2 diabetes: a randomized, double-blind, placebo-controlled study. *Diabetes Obes Metab* 2: 105-112.
